# Ethyl 5,5-dichloro-3-(4-chloro­phen­yl)-3a-methyl-4a-phenyl-3a,4,4a,5-tetra­hydro-3*H*-aziridino[2,1-*d*][1,2,4]triazolo[4,3-*a*][1,5]benzodiazepine-1-carboxyl­ate

**DOI:** 10.1107/S1600536811014115

**Published:** 2011-04-29

**Authors:** Aicha Boudina, Abdesselam Baouid, Mohamed Driss, El Hassane Soumhi

**Affiliations:** a Equipe de Chimie des Hétérocycles et Valorisation des Extraits des Plantes, Faculté des Sciences-Semlalia, Université Cadi Ayyad, Bd Abdelkrim Khattabi, BP 2390, 40001 Marrakech, Morocco; b Laboratoire de Matériaux et Cristallochimie, Faculté des Sciences de Tunis, Université de Tunis ElManar, 2092 ElManar II Tunis, Tunisia; c Equipe de Chimie des Matériaux et de l’Environnement, FSTG–Marrakech, Université Cadi Ayyad, Bd. Abdelkrim Khattabi, BP 549, Marrakech, Morocco

## Abstract

In the title compound, C_27_H_23_Cl_3_N_4_O_2_, the seven-membered diazepine ring adopts a boat conformation. The triazole ring makes dihedral angles of 17.24 (8) and 82.86 (8)°, respectively, with the chloro­benzene ring and the benzene ring of the benzodiazepine unit.

## Related literature

For background to benzodiazepine derivatives, see: Barltrop *et al.* (1959 ▶); El Hazazi *et al.* (2003 ▶); Sharp & Hamilton (1946 ▶). For related structures, see: Chiaroni *et al.* (1995 ▶); El Hazazi *et al.* (2000 ▶).
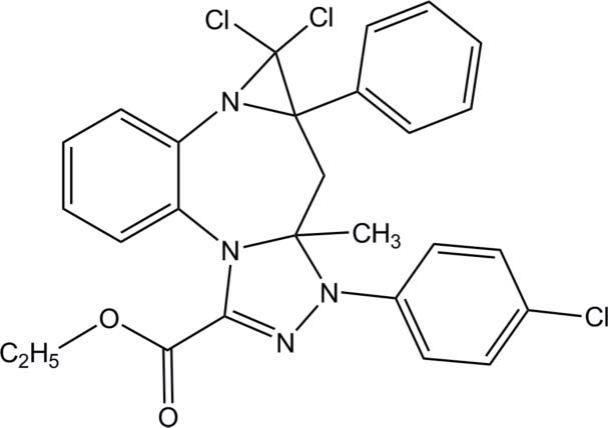


## Experimental

### 

#### Crystal data

C_27_H_23_Cl_3_N_4_O_2_
*M* *_r_* = 541.84
Triclinic, 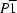

*a* = 9.679 (3) Å
*b* = 11.256 (3) Å
*c* = 12.661 (2) Å
α = 79.09 (2)°
β = 76.46 (2)°
γ = 73.04 (2)°
*V* = 1271.8 (6) Å^3^
*Z* = 2
Mo *K*α radiation
μ = 0.39 mm^−1^
*T* = 300 K
0.3 × 0.15 × 0.1 mm

#### Data collection

Enraf–Nonius CAD-4 diffractometer
6860 measured reflections
5536 independent reflections
4616 reflections with *I* > 2σ(*I*)
*R*_int_ = 0.010
2 standard reflections every 60 min  intensity decay: 1.0%

#### Refinement

*R*[*F* ^2^ > 2σ(*F* ^2^)] = 0.036
*wR*(*F* ^2^) = 0.101
*S* = 1.05
5536 reflections
327 parameters
H-atom parameters constrained
Δρ_max_ = 0.24 e Å^−3^
Δρ_min_ = −0.33 e Å^−3^

### 

Data collection: *CAD-4 EXPRESS* (Enraf–Nonius, 1989 ▶); cell refinement: *CAD-4 EXPRESS*; data reduction: *MolEN* (Fair, 1990 ▶); program(s) used to solve structure: *SHELXS97* (Sheldrick, 2008 ▶); program(s) used to refine structure: *SHELXL97* (Sheldrick, 2008 ▶); molecular graphics: *ORTEP-3 for Windows* (Farrugia, 1997 ▶); software used to prepare material for publication: *WinGX* (Farrugia, 1999 ▶).

## Supplementary Material

Crystal structure: contains datablocks I, global. DOI: 10.1107/S1600536811014115/is2697sup1.cif

Structure factors: contains datablocks I. DOI: 10.1107/S1600536811014115/is2697Isup2.hkl

Additional supplementary materials:  crystallographic information; 3D view; checkCIF report

## Figures and Tables

**Fig. 1.**
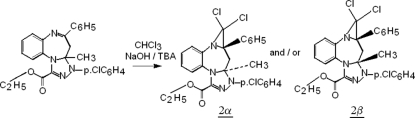
The reaction scheme of the title compound

**Fig. 2.**
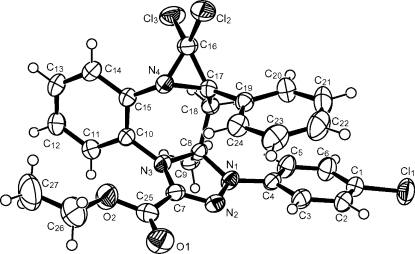
The molecular structure of the title compound, with 50% probability ellipsoids.

## supplementary crystallographic information

### Comment

In order to develop work carried out before in our laboratory we were interested in the synthesis of new derivatives benzodiazepinic (El Hazazi *et al.*, 2003). These reactions are either of the reactions of cycloadditions [2 + 1] realising generated carbenes *in situ* or reactions of transfer of methelyne.

In the present work, we report the synthesis of new benzodiazepine derivatives *via* addition of dichlorocarbene to [1,2,4]triazolo[4,3-*a*][1,5]benzodiazepine obtained stereospecifically by the addition of nitrilimines (Sharp *et al.*, 1946) on 1,5-benzodiazepine (Barltrop *et al.*, 1959).

Dichloroazacyclopropanation of [1,2,4]triazolo[4,3-*a*][1,5]benzodiazepine occurs readily under phase transfer catalysis conditions (liquid-liquid) with chloroform, aqueous sodium hydroxide and benzyltriethylammonium chloride (TBA-Cl) to give the corresponding bichloroadduct 2 (Fig. 1). Thus, the reaction of [1,2,4]triazolo[4,3-*a*][1,5]benzodiazepine 1 with dichlorocarbene in these conditions produce gem-dichloroaziridino[2,1-*d*][1,2,4] triazolo[4,3-*a*][1,5]benzodiazepine 2 in good yield.

The crystallographic study made it possible to determine the stereochemistry of the product 2. The crystalline structure confirms that the condensation of dichlorocarbene is carried out on double bond C=N substituted by the phenyl and shows that the product 2α obtained is of *trans* relative stereochemistry (Fig. 2). The main geometric features of this group are in good agreement it those observed in similar compound (Chiaroni *et al.*, 1995; El Hazazi *et al.*, 2000).

### Experimental

[1,2,4]Triazolo[4,3-*a*][1,5]benzodiazepine 1 (0.65 mm l) in 2 ml of chloroform were stirred with 2 ml of aqueous 50% NaOH solution and a catalytic amount of triethylbenzylammonium chloride (TBA-Cl). After 4 h the mixture was poured into 5 ml of water and extracted with ether. The organic phase was then dried over anhydrous sodium sulfate and the solvent was removed under reduced pressure. The crude product was chromatographied on a silica gel column (eluent: hexane/ethyl acetate 95/5) and recrystallized from ethanol/chloroform to give a compound 2α

The observation to be noted is that the condensation of dichlorocarbene to [1,2,4]triazolo[4,3-*a*][1,5]benzodiazepine is streospecific. The structure elucidation of the compound 2 was determinate on spectral data (^1^H NMR, ^13^C NMR and mass spectroscopy). The compound revealed in their spectra of mass the molecular peak located at m/*z* = 541 compatible with their empirical formula. The NMR spectrum of this product shows that the decalage of the chemical shifts of different grouping from monoadduct. In the ^13^C NMR spectrum of compound, we remarked the absence of the signals attributed to the double bond C5=N6 of cycle diazepinic. The ^13^C NMR spectrum of product was consistent with the presence of only one diasterioisomer. These spectral analyses do not enable us to determine relative stereochemistry of the aziridino[2,1-*d*][1,2,4]]triazolo[4,3-*a*][1,5]benzodiazepine (*trans* 2α or *cis* 2β).

### Refinement

All H atoms were located in a difference map and then refined using a riding model, with C—H = 0.96 Å and *U*_iso_(H) = 1.2*U*_eq_(C) for CH_3_, C—H = 0.97 Å and *U*_iso_(H) =1.2*U*_eq_(C) for CH_2_, and C—H = 0.93 Å and *U*_iso_(H) =1.2*U*_eq_(C) for CH.

### Crystal data

**Table:**

C_27_H_23_Cl_3_N_4_O_2_	*Z* = 2
*M**_r_* = 541.84	*F*(000) = 560
Triclinic, *P*1	*D*_x_ = 1.415 Mg m^−^^3^
Hall symbol: -P 1	Mo *K*α radiation, λ = 0.71073 Å
*a* = 9.679 (3) Å	Cell parameters from 25 reflections
*b* = 11.256 (3) Å	θ = 10–15°
*c* = 12.661 (2) Å	µ = 0.39 mm^−^^1^
α = 79.09 (2)°	*T* = 300 K
β = 76.46 (2)°	Prism, yellow
γ = 73.04 (2)°	0.3 × 0.15 × 0.1 mm
*V* = 1271.8 (6) Å^3^	

### Data collection

**Table:**

Enraf–Nonius CAD-4 diffractometer	*R*_int_ = 0.010
Radiation source: fine-focus sealed tube	θ_max_ = 27.0°, θ_min_ = 2.2°
graphite	*h* = −12→2
ω/2θ scans	*k* = −14→14
6860 measured reflections	*l* = −16→16
5536 independent reflections	2 standard reflections every 60 min
4616 reflections with *I* > 2σ(*I*)	intensity decay: 1.0%

### Refinement

**Table:**

Refinement on *F*^2^	Primary atom site location: structure-invariant direct methods
Least-squares matrix: full	Secondary atom site location: difference Fourier map
*R*[*F*^2^ > 2σ(*F*^2^)] = 0.036	Hydrogen site location: difference Fourier map
*wR*(*F*^2^) = 0.101	H-atom parameters constrained
*S* = 1.05	*w* = 1/[σ^2^(*F*_o_^2^) + (0.0518*P*)^2^ + 0.3226*P*] where *P* = (*F*_o_^2^ + 2*F*_c_^2^)/3
5536 reflections	(Δ/σ)_max_ = 0.001
327 parameters	Δρ_max_ = 0.24 e Å^−^^3^
0 restraints	Δρ_min_ = −0.33 e Å^−^^3^

### Special details

**Table:**

Geometry. All e.s.d.'s (except the e.s.d. in the dihedral angle between two l.s. planes) are estimated using the full covariance matrix. The cell e.s.d.'s are taken into account individually in the estimation of e.s.d.'s in distances, angles and torsion angles; correlations between e.s.d.'s in cell parameters are only used when they are defined by crystal symmetry. An approximate (isotropic) treatment of cell e.s.d.'s is used for estimating e.s.d.'s involving l.s. planes.
Refinement. Refinement of *F*^2^ against ALL reflections. The weighted *R*-factor *wR* and goodness of fit *S* are based on *F*^2^, conventional *R*-factors *R* are based on *F*, with *F* set to zero for negative *F*^2^. The threshold expression of *F*^2^ > σ(*F*^2^) is used only for calculating *R*-factors(gt) *etc*. and is not relevant to the choice of reflections for refinement. *R*-factors based on *F*^2^ are statistically about twice as large as those based on *F*, and *R*- factors based on ALL data will be even larger.

### Fractional atomic coordinates and isotropic or equivalent isotropic displacement parameters (Å^2^)

**Table:**

	*x*	*y*	*z*	*U*_iso_*/*U*_eq_	
Cl1	1.31467 (5)	−0.06197 (4)	0.57617 (5)	0.06714 (16)	
Cl2	0.74695 (5)	0.33836 (4)	0.03031 (4)	0.05465 (13)	
Cl3	0.50575 (5)	0.37629 (5)	0.21522 (4)	0.05877 (14)	
O1	1.09072 (14)	0.71899 (13)	0.20132 (12)	0.0619 (3)	
O2	0.85011 (15)	0.79523 (12)	0.19906 (12)	0.0638 (4)	
N1	0.94972 (12)	0.42660 (12)	0.40621 (10)	0.0369 (3)	
N2	1.02302 (13)	0.51079 (12)	0.34036 (10)	0.0366 (3)	
N3	0.78121 (13)	0.58997 (11)	0.34267 (10)	0.0354 (3)	
N4	0.72403 (13)	0.50553 (11)	0.16401 (10)	0.0361 (3)	
C1	1.20506 (17)	0.07966 (15)	0.52295 (14)	0.0444 (3)	
C2	1.26875 (17)	0.15749 (15)	0.44036 (15)	0.0459 (4)	
H2	1.3689	0.1335	0.4116	0.055*	
C3	1.18305 (16)	0.27115 (15)	0.40070 (13)	0.0413 (3)	
H3	1.2258	0.3230	0.3444	0.050*	
C4	1.03279 (15)	0.30883 (13)	0.44432 (11)	0.0346 (3)	
C5	0.97014 (17)	0.22829 (16)	0.52598 (14)	0.0470 (4)	
H5	0.8699	0.2514	0.5548	0.056*	
C6	1.05614 (19)	0.11349 (16)	0.56493 (15)	0.0507 (4)	
H6	1.0135	0.0596	0.6191	0.061*	
C7	0.92343 (15)	0.60263 (13)	0.30379 (11)	0.0345 (3)	
C8	0.78887 (14)	0.46551 (13)	0.40868 (11)	0.0322 (3)	
C9	0.69700 (16)	0.47994 (15)	0.52411 (12)	0.0405 (3)	
H9A	0.7524	0.5014	0.5681	0.049*	
H9B	0.6731	0.4025	0.5568	0.049*	
H9C	0.6079	0.5450	0.5197	0.049*	
C10	0.65238 (15)	0.65391 (13)	0.29762 (12)	0.0355 (3)	
C11	0.55573 (18)	0.75943 (15)	0.34035 (15)	0.0464 (4)	
H11	0.5753	0.7887	0.3978	0.056*	
C12	0.43014 (19)	0.82118 (16)	0.29742 (17)	0.0555 (4)	
H12	0.3658	0.8918	0.3259	0.067*	
C13	0.40124 (19)	0.77727 (16)	0.21230 (17)	0.0567 (5)	
H13	0.3171	0.8187	0.1837	0.068*	
C14	0.49615 (18)	0.67216 (16)	0.16904 (15)	0.0492 (4)	
H14	0.4760	0.6436	0.1115	0.059*	
C15	0.62242 (15)	0.60913 (13)	0.21222 (12)	0.0366 (3)	
C16	0.68332 (17)	0.39468 (14)	0.15834 (13)	0.0403 (3)	
C17	0.78959 (15)	0.38713 (12)	0.23111 (11)	0.0333 (3)	
C18	0.73573 (15)	0.37907 (13)	0.35398 (11)	0.0336 (3)	
H18A	0.7696	0.2931	0.3866	0.040*	
H18B	0.6290	0.4013	0.3692	0.040*	
C19	0.94952 (15)	0.32550 (13)	0.19097 (11)	0.0351 (3)	
C20	0.99906 (19)	0.19656 (15)	0.21963 (14)	0.0483 (4)	
H20	0.9342	0.1518	0.2625	0.058*	
C21	1.1452 (2)	0.13472 (18)	0.18427 (17)	0.0628 (5)	
H21	1.1776	0.0486	0.2031	0.075*	
C22	1.2422 (2)	0.2009 (2)	0.12121 (17)	0.0644 (5)	
H22	1.3402	0.1597	0.0985	0.077*	
C23	1.19335 (19)	0.3282 (2)	0.09202 (15)	0.0570 (4)	
H23	1.2588	0.3726	0.0494	0.068*	
C24	1.04672 (17)	0.39071 (15)	0.12584 (12)	0.0427 (3)	
H24	1.0141	0.4763	0.1046	0.051*	
C25	0.96658 (18)	0.71092 (15)	0.22958 (13)	0.0422 (3)	
C26	0.8773 (3)	0.9023 (2)	0.1207 (2)	0.0818 (7)	
H26A	0.9327	0.8752	0.0512	0.098*	
H26B	0.9332	0.9448	0.1478	0.098*	
C27	0.7316 (4)	0.9875 (2)	0.1068 (3)	0.0985 (9)	
H27A	0.6806	1.0179	0.1751	0.118*	
H27B	0.6750	0.9428	0.0844	0.118*	
H27C	0.7451	1.0570	0.0520	0.118*	

### Atomic displacement parameters (Å^2^)

**Table:**

	*U*^11^	*U*^22^	*U*^33^	*U*^12^	*U*^13^	*U*^23^
Cl1	0.0493 (3)	0.0441 (2)	0.0967 (4)	0.00124 (19)	−0.0208 (2)	0.0058 (2)
Cl2	0.0623 (3)	0.0590 (3)	0.0487 (2)	−0.0108 (2)	−0.01682 (19)	−0.02154 (19)
Cl3	0.0403 (2)	0.0739 (3)	0.0728 (3)	−0.0232 (2)	−0.0127 (2)	−0.0197 (2)
O1	0.0495 (7)	0.0679 (8)	0.0696 (8)	−0.0300 (6)	−0.0053 (6)	0.0043 (7)
O2	0.0588 (8)	0.0488 (7)	0.0836 (9)	−0.0227 (6)	−0.0257 (7)	0.0213 (6)
N1	0.0249 (5)	0.0414 (6)	0.0421 (6)	−0.0082 (5)	−0.0074 (5)	0.0008 (5)
N2	0.0305 (6)	0.0423 (6)	0.0388 (6)	−0.0127 (5)	−0.0072 (5)	−0.0041 (5)
N3	0.0284 (6)	0.0341 (6)	0.0449 (7)	−0.0084 (5)	−0.0116 (5)	−0.0022 (5)
N4	0.0338 (6)	0.0364 (6)	0.0382 (6)	−0.0048 (5)	−0.0115 (5)	−0.0060 (5)
C1	0.0385 (8)	0.0380 (8)	0.0562 (9)	−0.0028 (6)	−0.0162 (7)	−0.0068 (7)
C2	0.0280 (7)	0.0452 (8)	0.0620 (10)	−0.0033 (6)	−0.0075 (7)	−0.0117 (7)
C3	0.0298 (7)	0.0439 (8)	0.0488 (8)	−0.0096 (6)	−0.0050 (6)	−0.0060 (6)
C4	0.0282 (6)	0.0395 (7)	0.0364 (7)	−0.0055 (5)	−0.0094 (5)	−0.0066 (6)
C5	0.0310 (7)	0.0518 (9)	0.0487 (9)	−0.0049 (7)	−0.0032 (6)	0.0020 (7)
C6	0.0421 (9)	0.0482 (9)	0.0530 (9)	−0.0075 (7)	−0.0071 (7)	0.0056 (7)
C7	0.0316 (7)	0.0377 (7)	0.0377 (7)	−0.0113 (6)	−0.0083 (6)	−0.0078 (6)
C8	0.0250 (6)	0.0347 (7)	0.0359 (7)	−0.0057 (5)	−0.0073 (5)	−0.0038 (5)
C9	0.0301 (7)	0.0508 (9)	0.0400 (8)	−0.0079 (6)	−0.0041 (6)	−0.0116 (6)
C10	0.0286 (6)	0.0324 (7)	0.0460 (8)	−0.0061 (5)	−0.0114 (6)	−0.0038 (6)
C11	0.0435 (8)	0.0382 (8)	0.0587 (10)	−0.0036 (6)	−0.0152 (7)	−0.0137 (7)
C12	0.0436 (9)	0.0403 (8)	0.0784 (12)	0.0061 (7)	−0.0183 (8)	−0.0160 (8)
C13	0.0401 (9)	0.0470 (9)	0.0814 (13)	0.0042 (7)	−0.0291 (9)	−0.0072 (9)
C14	0.0435 (9)	0.0478 (9)	0.0594 (10)	−0.0026 (7)	−0.0256 (8)	−0.0093 (7)
C15	0.0307 (7)	0.0343 (7)	0.0444 (8)	−0.0049 (5)	−0.0102 (6)	−0.0056 (6)
C16	0.0369 (7)	0.0444 (8)	0.0428 (8)	−0.0098 (6)	−0.0095 (6)	−0.0123 (6)
C17	0.0313 (7)	0.0313 (7)	0.0374 (7)	−0.0071 (5)	−0.0073 (5)	−0.0053 (5)
C18	0.0293 (6)	0.0347 (7)	0.0371 (7)	−0.0098 (5)	−0.0047 (5)	−0.0046 (5)
C19	0.0335 (7)	0.0362 (7)	0.0340 (7)	−0.0049 (6)	−0.0063 (5)	−0.0077 (5)
C20	0.0481 (9)	0.0363 (8)	0.0540 (9)	−0.0037 (7)	−0.0067 (7)	−0.0052 (7)
C21	0.0583 (11)	0.0456 (9)	0.0701 (12)	0.0122 (8)	−0.0128 (9)	−0.0124 (9)
C22	0.0386 (9)	0.0769 (13)	0.0635 (12)	0.0086 (9)	−0.0027 (8)	−0.0211 (10)
C23	0.0388 (9)	0.0767 (13)	0.0500 (10)	−0.0136 (8)	0.0024 (7)	−0.0103 (9)
C24	0.0395 (8)	0.0464 (8)	0.0393 (8)	−0.0090 (7)	−0.0050 (6)	−0.0052 (6)
C25	0.0466 (9)	0.0430 (8)	0.0424 (8)	−0.0187 (7)	−0.0096 (7)	−0.0062 (6)
C26	0.0976 (18)	0.0566 (12)	0.0926 (17)	−0.0362 (12)	−0.0307 (14)	0.0282 (11)
C27	0.129 (2)	0.0516 (12)	0.104 (2)	−0.0106 (14)	−0.0397 (18)	0.0166 (13)

### Geometric parameters (Å, °)

**Table:**

Cl1—C1	1.7500 (17)	C10—C11	1.392 (2)
Cl2—C16	1.7614 (16)	C10—C15	1.395 (2)
Cl3—C16	1.7570 (17)	C11—C12	1.389 (2)
O1—C25	1.196 (2)	C11—H11	0.9300
O2—C25	1.327 (2)	C12—C13	1.381 (3)
O2—C26	1.455 (2)	C12—H12	0.9300
N1—N2	1.3830 (17)	C13—C14	1.385 (2)
N1—C4	1.3979 (18)	C13—H13	0.9300
N1—C8	1.4837 (17)	C14—C15	1.399 (2)
N2—C7	1.2878 (19)	C14—H14	0.9300
N3—C7	1.3886 (18)	C16—C17	1.509 (2)
N3—C10	1.4326 (18)	C17—C19	1.5081 (19)
N3—C8	1.4804 (18)	C17—C18	1.5148 (19)
N4—C15	1.4209 (19)	C18—H18A	0.9700
N4—C16	1.4322 (19)	C18—H18B	0.9700
N4—C17	1.4936 (18)	C19—C24	1.384 (2)
C1—C6	1.379 (2)	C19—C20	1.394 (2)
C1—C2	1.381 (2)	C20—C21	1.390 (3)
C2—C3	1.382 (2)	C20—H20	0.9300
C2—H2	0.9300	C21—C22	1.380 (3)
C3—C4	1.397 (2)	C21—H21	0.9300
C3—H3	0.9300	C22—C23	1.378 (3)
C4—C5	1.390 (2)	C22—H22	0.9300
C5—C6	1.389 (2)	C23—C24	1.393 (2)
C5—H5	0.9300	C23—H23	0.9300
C6—H6	0.9300	C24—H24	0.9300
C7—C25	1.491 (2)	C26—C27	1.484 (4)
C8—C9	1.533 (2)	C26—H26A	0.9700
C8—C18	1.5506 (19)	C26—H26B	0.9700
C9—H9A	0.9600	C27—H27A	0.9600
C9—H9B	0.9600	C27—H27B	0.9600
C9—H9C	0.9600	C27—H27C	0.9600
			
C25—O2—C26	117.07 (16)	C13—C14—H14	120.1
N2—N1—C4	118.46 (11)	C15—C14—H14	120.1
N2—N1—C8	113.24 (11)	C10—C15—C14	119.52 (14)
C4—N1—C8	127.07 (12)	C10—C15—N4	120.50 (12)
C7—N2—N1	106.12 (12)	C14—C15—N4	119.84 (14)
C7—N3—C10	127.78 (12)	N4—C16—C17	60.97 (9)
C7—N3—C8	108.57 (11)	N4—C16—Cl3	121.86 (11)
C10—N3—C8	119.40 (11)	C17—C16—Cl3	120.64 (11)
C15—N4—C16	122.29 (12)	N4—C16—Cl2	114.44 (11)
C15—N4—C17	122.26 (12)	C17—C16—Cl2	120.61 (11)
C16—N4—C17	62.05 (9)	Cl3—C16—Cl2	110.44 (8)
C6—C1—C2	120.55 (15)	N4—C17—C19	116.19 (12)
C6—C1—Cl1	119.73 (14)	N4—C17—C16	56.97 (9)
C2—C1—Cl1	119.71 (12)	C19—C17—C16	117.06 (12)
C1—C2—C3	119.74 (14)	N4—C17—C18	116.74 (11)
C1—C2—H2	120.1	C19—C17—C18	117.07 (12)
C3—C2—H2	120.1	C16—C17—C18	119.10 (12)
C2—C3—C4	120.61 (15)	C17—C18—C8	113.50 (11)
C2—C3—H3	119.7	C17—C18—H18A	108.9
C4—C3—H3	119.7	C8—C18—H18A	108.9
C5—C4—C3	118.81 (14)	C17—C18—H18B	108.9
C5—C4—N1	121.63 (13)	C8—C18—H18B	108.9
C3—C4—N1	119.55 (13)	H18A—C18—H18B	107.7
C6—C5—C4	120.48 (14)	C24—C19—C20	119.32 (14)
C6—C5—H5	119.8	C24—C19—C17	122.92 (13)
C4—C5—H5	119.8	C20—C19—C17	117.74 (14)
C1—C6—C5	119.76 (16)	C21—C20—C19	120.20 (17)
C1—C6—H6	120.1	C21—C20—H20	119.9
C5—C6—H6	120.1	C19—C20—H20	119.9
N2—C7—N3	113.87 (13)	C22—C21—C20	120.11 (17)
N2—C7—C25	119.72 (13)	C22—C21—H21	119.9
N3—C7—C25	126.39 (13)	C20—C21—H21	119.9
N3—C8—N1	97.86 (10)	C23—C22—C21	119.85 (17)
N3—C8—C9	110.22 (12)	C23—C22—H22	120.1
N1—C8—C9	113.22 (11)	C21—C22—H22	120.1
N3—C8—C18	111.80 (11)	C22—C23—C24	120.47 (18)
N1—C8—C18	113.43 (11)	C22—C23—H23	119.8
C9—C8—C18	109.85 (11)	C24—C23—H23	119.8
C8—C9—H9A	109.5	C19—C24—C23	120.03 (16)
C8—C9—H9B	109.5	C19—C24—H24	120.0
H9A—C9—H9B	109.5	C23—C24—H24	120.0
C8—C9—H9C	109.5	O1—C25—O2	125.08 (15)
H9A—C9—H9C	109.5	O1—C25—C7	123.70 (16)
H9B—C9—H9C	109.5	O2—C25—C7	111.22 (13)
C11—C10—C15	119.98 (13)	O2—C26—C27	107.1 (2)
C11—C10—N3	119.99 (14)	O2—C26—H26A	110.3
C15—C10—N3	120.01 (13)	C27—C26—H26A	110.3
C12—C11—C10	120.18 (16)	O2—C26—H26B	110.3
C12—C11—H11	119.9	C27—C26—H26B	110.3
C10—C11—H11	119.9	H26A—C26—H26B	108.6
C13—C12—C11	119.76 (16)	C26—C27—H27A	109.5
C13—C12—H12	120.1	C26—C27—H27B	109.5
C11—C12—H12	120.1	H27A—C27—H27B	109.5
C12—C13—C14	120.77 (15)	C26—C27—H27C	109.5
C12—C13—H13	119.6	H27A—C27—H27C	109.5
C14—C13—H13	119.6	H27B—C27—H27C	109.5
C13—C14—C15	119.79 (16)		
			
C4—N1—N2—C7	170.77 (12)	C16—N4—C15—C10	123.80 (15)
C8—N1—N2—C7	2.53 (16)	C17—N4—C15—C10	48.67 (19)
C6—C1—C2—C3	1.1 (3)	C16—N4—C15—C14	−60.6 (2)
Cl1—C1—C2—C3	−177.96 (13)	C17—N4—C15—C14	−135.70 (15)
C1—C2—C3—C4	0.8 (2)	C15—N4—C16—C17	−112.30 (14)
C2—C3—C4—C5	−2.0 (2)	C15—N4—C16—Cl3	−2.34 (19)
C2—C3—C4—N1	177.17 (14)	C17—N4—C16—Cl3	109.96 (14)
N2—N1—C4—C5	168.12 (14)	C15—N4—C16—Cl2	134.90 (12)
C8—N1—C4—C5	−25.5 (2)	C17—N4—C16—Cl2	−112.80 (12)
N2—N1—C4—C3	−11.0 (2)	C15—N4—C17—C19	−141.09 (13)
C8—N1—C4—C3	155.43 (14)	C16—N4—C17—C19	106.56 (14)
C3—C4—C5—C6	1.3 (2)	C15—N4—C17—C16	112.35 (15)
N1—C4—C5—C6	−177.85 (15)	C15—N4—C17—C18	3.56 (18)
C2—C1—C6—C5	−1.8 (3)	C16—N4—C17—C18	−108.79 (14)
Cl1—C1—C6—C5	177.27 (14)	Cl3—C16—C17—N4	−111.90 (13)
C4—C5—C6—C1	0.6 (3)	Cl2—C16—C17—N4	102.80 (13)
N1—N2—C7—N3	1.62 (16)	N4—C16—C17—C19	−105.01 (14)
N1—N2—C7—C25	179.78 (12)	Cl3—C16—C17—C19	143.09 (12)
C10—N3—C7—N2	−161.35 (14)	Cl2—C16—C17—C19	−2.21 (18)
C8—N3—C7—N2	−5.08 (17)	N4—C16—C17—C18	104.62 (14)
C10—N3—C7—C25	20.6 (2)	Cl3—C16—C17—C18	−7.28 (18)
C8—N3—C7—C25	176.90 (13)	Cl2—C16—C17—C18	−152.58 (11)
C7—N3—C8—N1	5.69 (13)	N4—C17—C18—C8	−70.59 (15)
C10—N3—C8—N1	164.27 (12)	C19—C17—C18—C8	73.75 (15)
C7—N3—C8—C9	124.04 (12)	C16—C17—C18—C8	−135.88 (13)
C10—N3—C8—C9	−77.37 (15)	N3—C8—C18—C17	41.51 (15)
C7—N3—C8—C18	−113.48 (12)	N1—C8—C18—C17	−67.98 (15)
C10—N3—C8—C18	45.10 (16)	C9—C8—C18—C17	164.20 (12)
N2—N1—C8—N3	−5.08 (14)	N4—C17—C19—C24	24.5 (2)
C4—N1—C8—N3	−172.10 (13)	C16—C17—C19—C24	88.97 (18)
N2—N1—C8—C9	−121.11 (13)	C18—C17—C19—C24	−120.05 (15)
C4—N1—C8—C9	71.87 (18)	N4—C17—C19—C20	−154.13 (13)
N2—N1—C8—C18	112.84 (13)	C16—C17—C19—C20	−89.64 (17)
C4—N1—C8—C18	−54.18 (18)	C18—C17—C19—C20	61.34 (18)
C7—N3—C10—C11	−97.33 (19)	C24—C19—C20—C21	0.9 (3)
C8—N3—C10—C11	108.64 (16)	C17—C19—C20—C21	179.56 (16)
C7—N3—C10—C15	83.81 (19)	C19—C20—C21—C22	0.5 (3)
C8—N3—C10—C15	−70.22 (18)	C20—C21—C22—C23	−1.0 (3)
C15—C10—C11—C12	−0.7 (3)	C21—C22—C23—C24	0.1 (3)
N3—C10—C11—C12	−179.54 (15)	C20—C19—C24—C23	−1.7 (2)
C10—C11—C12—C13	0.2 (3)	C17—C19—C24—C23	179.67 (15)
C11—C12—C13—C14	0.0 (3)	C22—C23—C24—C19	1.2 (3)
C12—C13—C14—C15	0.3 (3)	C26—O2—C25—O1	3.4 (3)
C11—C10—C15—C14	1.0 (2)	C26—O2—C25—C7	−176.14 (17)
N3—C10—C15—C14	179.86 (14)	N2—C7—C25—O1	−0.4 (2)
C11—C10—C15—N4	176.65 (14)	N3—C7—C25—O1	177.46 (15)
N3—C10—C15—N4	−4.5 (2)	N2—C7—C25—O2	179.06 (14)
C13—C14—C15—C10	−0.8 (3)	N3—C7—C25—O2	−3.0 (2)
C13—C14—C15—N4	−176.51 (16)	C25—O2—C26—C27	−175.3 (2)
